# Researching the application of virtual reality in medical education: one-year follow-up of a randomized trial

**DOI:** 10.1186/s12909-022-03992-6

**Published:** 2023-01-03

**Authors:** Wenyi Gan, Tsz-Ngai Mok, Junyuan Chen, Guorong She, Zhengang Zha, Huajun Wang, Hua Li, Jieruo Li, Xiaofei Zheng

**Affiliations:** 1 Institute of Orthopedics Diseases and Center for Joint Surgery and Sports Medicine The First Affiliated Hospital of Jinan University, Guangzhou, China; 2 Department of orthopedics, General Hospital of Chinese PLA, No 28 Fuxing Road, 100853 Beijing, China

**Keywords:** Virtual reality, Medical Education, Nonlinear Dynamics, Internship and residency

## Abstract

**Background:**

Compared with traditional tendon repair teaching methods, using a virtual reality (VR) simulator to teach tendon suturing can significantly improve medical students’ exercise time, operation flow and operation knowledge. At present, the purpose of this study is to explore the long-term influence of VR simulator teaching on the practice performance of medical students.

**Method:**

This is a one-year long-term follow-up study of a randomized controlled study. A total of 117 participants who completed the initial study were invited to participate in the follow-up study. Participants in the VR group and the control group were required to complete a questionnaire developed by the authors and the teachers in the teaching and research department and to provide their surgical internship scores and Objective Structure Clinical Examination(OSCE) graduation scores.

**Results:**

Of the 117 invitees, 108 completed the follow-up. The answers to the questions about career choice and study habits were more positive in the VR group than in the control group (*p* < 0.05). The total score for clinical practice in the VR group was better than that in the control group, and the difference was statistically significant (*p* < 0.05). In the OSCE examination, the scores for physical examination, suturing and knotting and image reading were higher in the VR group than in the control group, and the difference was statistically significant (*p* < 0.05).

**Conclusion:**

The results of the one-year long-term follow-up indicated that compared with medical students experiencing the traditional teaching mode, those experiencing the VR teaching mode had more determined career pursuit and active willingness to learn, better evaluations from teachers in the process of surgical clinical practice, and better scores in physical examination, suturing and knotting and image reading in the OSCE examination. In the study of nonlinear dynamics to cultivate a good learning model for medical students, the VR teaching model is expected to become an effective and stable initial sensitive element.

**Trial registration:**

Chinese Clinical Trial Registry(25/05/2021, ChiCTR2100046648); http://www.chictr.org.cn/hvshowproject.aspx?id=90180.

**Supplementary Information:**

The online version contains supplementary material available at 10.1186/s12909-022-03992-6.

## Introduction

How to effectively improve the medical knowledge reserve ability of medical students and establish an effective relationship between professional theoretical study and clinical practice are two major problems currently facing global medical education [1].The medical knowledge system is huge, the content varies, and the learning cycle is long. It is difficult for medical students to establish a solid knowledge system and enhance their perceptual medical knowledge only through traditional two-dimensional learning. The traditional one-way input theoretical learning and autopsy learning mode cannot be individualized for specific students, and autopsy learning is limited by ethical issues and the number of available specimens, so it is difficult to maintain teaching quality [1, 2].

In recent years, virtual reality (VR) simulators have been widely used in the field of medical teaching and have achieved good results. Many hospitals are now using virtual reality to assist senior surgical residents in improving surgical technology, and this training has shown to enhance learning efficiency, knowledge transfer, and skill transfer [3]. It is because VR simulators can give prompt feedback to learners from visual and aural multisensory and multiangle viewpoints [4], which is also critical in clinical medical education [5]. According to the different input operations of the operator, they can formulate the corresponding processing results and output them in real time, which is also called real-time interaction [6]. This kind of immediate feedback can prompt the operators to change the learning process from passive to active and construct a relationship between visual feedback and psychological expectations in the learning process [7–9]. The improvement in learners’ three-dimensional imagination, active operation and exploration of learning skills can improve their knowledge reserve ability. According to the most recent comparative bibliometrics analysis, students may grasp the theoretical understanding of learning and improve the teaching impact with the use of 3D technology in the classroom [10]. However, this comprehension is still limited to the learning topic; there is no way to make a meaningful link to the real operation [10]. Compared with traditional teaching methods(or combining with 3D technology), this immersive learning method provided by VR technology can arouse students’ interest in learning, stimulate their motivation for active learning and build their individual medical knowledge reserve system [11, 12].

Sports [13], tourism [14], surgical simulation [15], rehabilitation counseling [16], simulated military operations [17], and education [18] are some of the current uses for virtual reality. A secure, consistent, and individualized learning environment may be offered via a VR learning platform [19]. Learners may fully express their subjective initiative on the VR platform and, through repeated efforts and exercises, enhance their grasp and cognition of a specific professional expertise. The prevalence of new COVID-19 has further encouraged the use of VR technology in the area of education [20, 21]. VR is now playing a bigger and bigger part in the educational world as online learning steadily replaces traditional classroom instruction[19]. The majority of study subjects in the medical area of VR have become physicians through clinical practice, however there is a dearth of research on the education of medical students at the undergraduate level. In addition to surgical technical supervision and tele-rehabilitation research demonstrating that VR may cure certain major illnesses, supporting VR instruction in undergraduate education is essential, since it is an essential component of VR's spread [22].

In our initial research, we pioneered the use of VR in undergraduate surgical Achilles tendon suture instruction and discovered that students in the experimental group were better able to grasp the procedure [18]. The high application cost is the main obstacle to popularizing VR education in various countries and regions. The rapid progress and iterative adaptation of modern digital technology is the inevitable trend of the development of science and technology [23]. With continuous changes in technology, in addition to enriching the diversity of VR teaching forms, reducing the application cost is an inevitable trend of science and technology products [23]. Even though the current application of VR simulation has some shortcomings, such as increasing the mental pressure on users and high cost [24, 25], integrating digital teaching into medical courses will be a long-term and continuous process. From the point of view of teaching purposes, VR simulation improves the quality and efficiency of education and is a supplement to traditional education rather than a substitute. During the COVID-19 epidemic, the need for remote education has skyrocketed, and VR applications in undergraduate education are also on the rise, particularly in medical education that calls for clinical learning experiences [16, 26]. In order to promote the use of VR in the field, we require high-quality long-term follow-up to look at the application value of VR training in undergraduate medical education.

Therefore, this study is a long-term follow-up study of the first clinical randomized trial to explore whether students in the VR experimental group could sum up their learning experience and had developed positive learning habits after experiencing VR immersion teaching compared with traditional teaching and whether they could define their future career planning, improve their clinical practice performance and improve their graduation examination scores during the one-year clinical practice study.

## Method

**Fig. 1:**
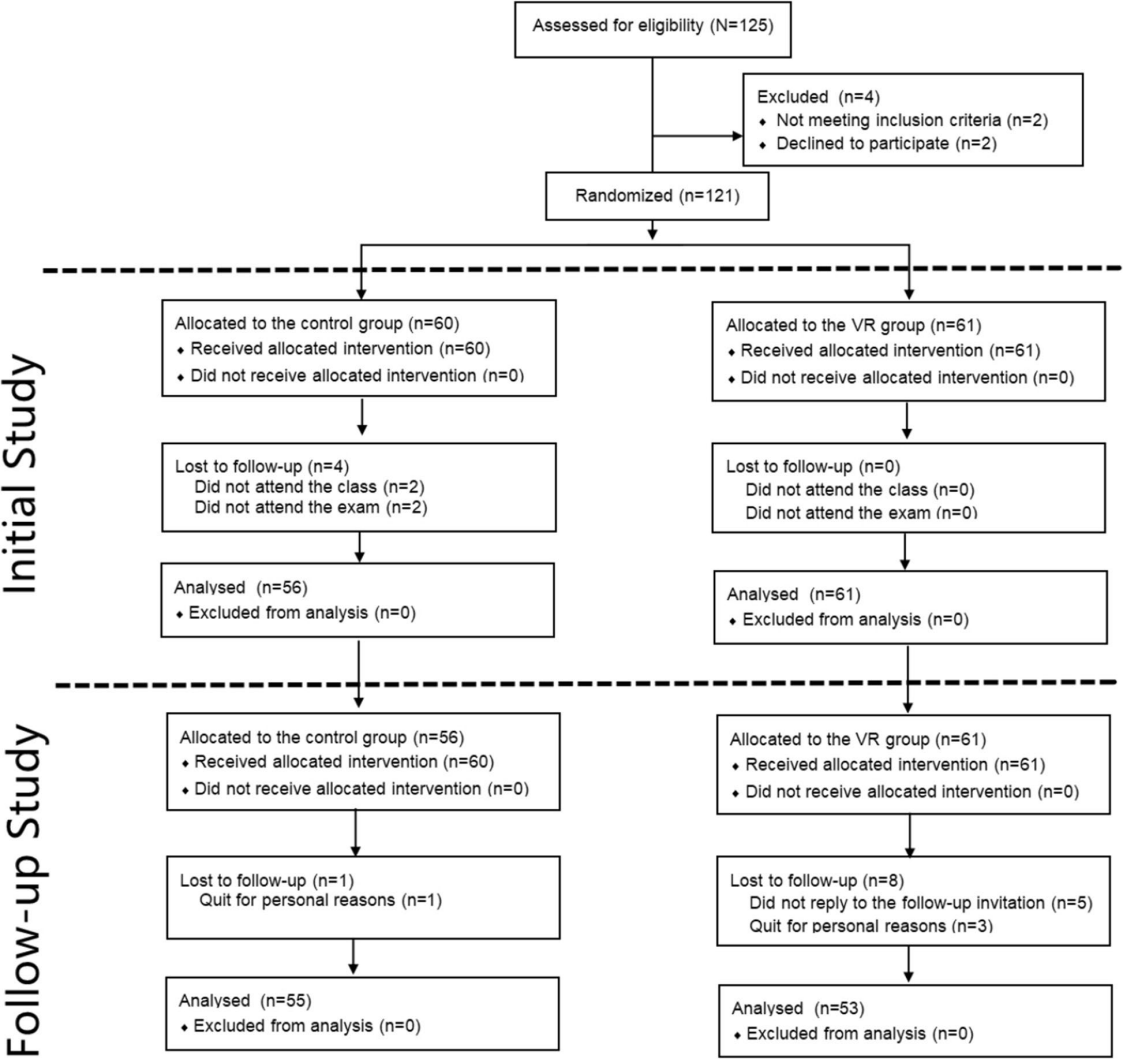
CONSORT 2010 flow diagram showing the study process. VR: virtual reality

A total of 117 fourth-year medical students participated in the initial trial and then participated in a one-year clinical internship under the teaching plan (Fig. 1) (25/05/2021,ChiCTR2100046648). The five-year clinical students of Jinan University do not receive directional assignments during the clinical practice but undergo an internship plan in 18 departments. They take the Objective Structure Clinical Examination (OSCE) after the internship, and those who pass the examination have finished their undergraduate studies. The two writers gathered participant scores from the student internship evaluation system and OSCE assessment system under the assumption that all participants had completed informed permission forms.

### Participants

We sent follow-up applications to 117 medical students who participated in the initial trial, and 108 responded and participated in the follow-up. The main purposes of the follow-up were to obtain the surgical internship scores and OSCE results of the participants and to have them complete a questionnaire.

#### Inclusion criteria

The participants who sign an informed consent form for this follow-up study had to meet the following requirements:

- 1. Complete the required course in the first trial and participate in the assessment of tendon suturing;
- 2. A one-year internship at a hospital is required.

#### Exclusion criteria

- 1. Failed to complete the course specified in the initial experiment;
- 2. Did not participate in the examination and evaluation of tendon suturing in the first trial;
- 3. Failed to complete the rotation of all clinical departments required by the internship within 1 years after the initial trial;
- 4. Low Grade Point Average (GPA) or personal reasons;

### Intervention

#### Initial study

The VR simulator used in this study was built by Orthopedic Surgery and Sports Medicine Center and Jinan University. SteamVR was the program used by all VR simulators (JinKe Lu). Each participant in this study must read all of the website's ideas in order to use the VR simulator approach. There are two variants (ie, PC and VR simulator versions). Students used the PC keyboard and mouse to learn about tendon repair. A 360-degree scenario is included in the PC edition to enhance the learning environment.

**Fig. 2:**
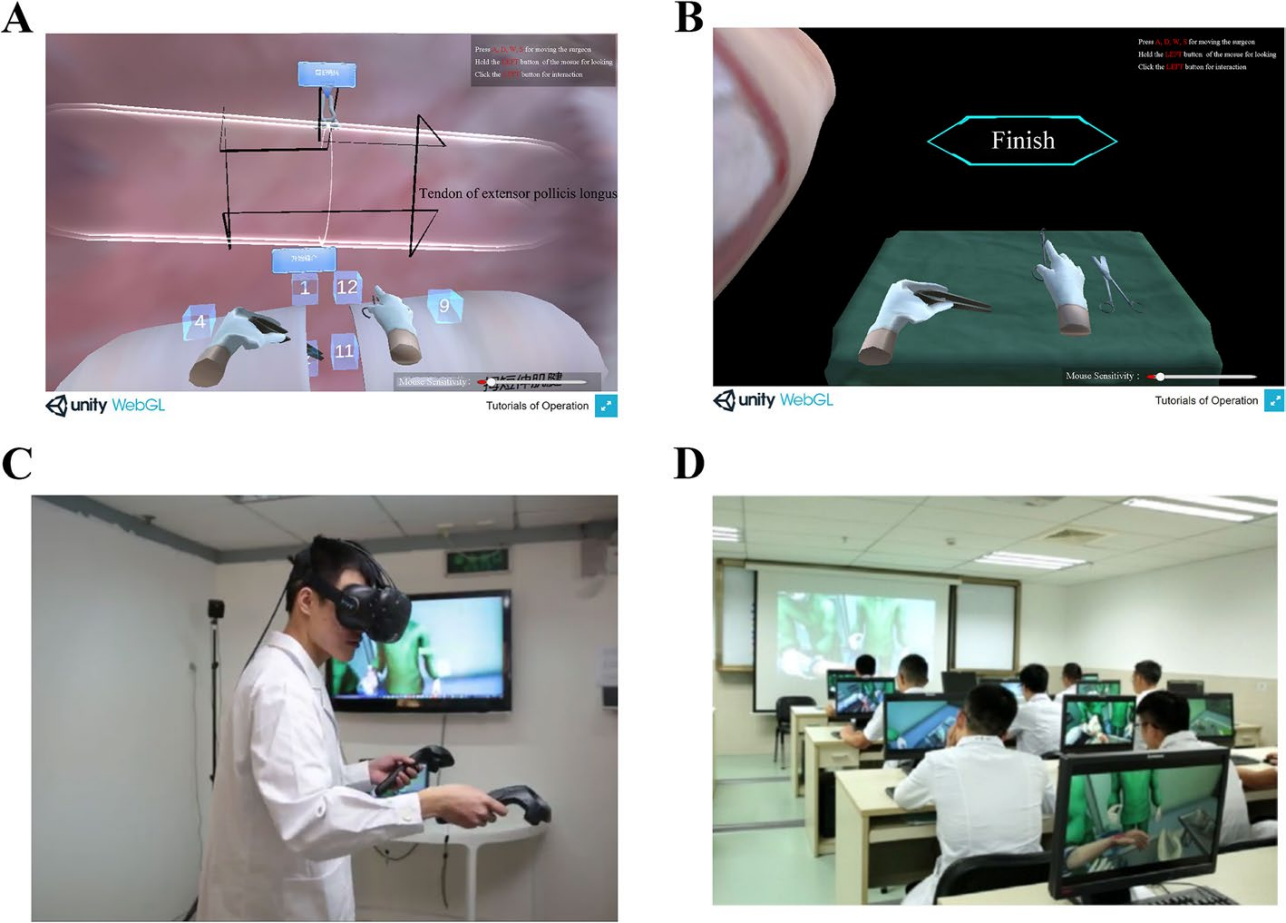
**A-C**: Students in the experimental group used VR simulator for tendon suture operation practice and examination; (**D**) Students in the control group used PowerPoint presentation to learn tendon suture operation

All participants had to attend 8 h of lectures and 6 h of practical classes for 2 weeks. During the lectures, participants learnt about traumatic orthopedic theory and tendon healing basics. This lecture used a PowerPoint presentation with drawings, photos, and step-by-step directions. The control group were given one hour to review the information (Fig. 2D). The VR group took the identical course as the control group, except for the guided PowerPoint review. Instead, they spent an hour in class practicing with VR simulators (both VR and PC) (Fig. 2A-C). The VR simulator focuses on each participant's tendon restoration performance (Supplementary Materials S1). The VR simulator has two modes of operation: practice and test. Students have to complete all essential learning in practice mode before moving on to evaluation mode. All training was done in class, and both groups got the same amount of practice time.

#### Follow-up study

The major duties were to distribute surveys and collect internship and OSCE scores, and we did not interfere in the follow-up research.

### Questionnaire design

To evaluate the long-term impact of using VR applications on participants’ surgical learning and career planning, our research team looked at the existing literature and identified the key topics, such as interns' attitudes about internship work [27, 28], their passion for work [28], their capacity to actively locate and solve problems [27, 29], their interpersonal skills at work [29], and their ability to plan their future career [30, 31]. The main author of this article convened with the teaching secretaries of the surgery clinical department of the First Affiliated Hospital of Jinan University (the rotation surgery departments of undergraduate clinical medical students are urology, gastrointestinal surgery, breast surgery, orthopedics, cardiac surgery and thoracic surgery) with the purpose of developing a targeted questionnaire survey. First, the authors and teaching secretaries designed the questionnaire questions; then, they discussed the contents of each topic within the group, modified the wording of each question, and finally voted on the questions to be included in the questionnaire. Questions that received more than 75% of the votes were included in the final questionnaire survey. The design of this questionnaire referred to the idea of the group recommended by the European League Against Rheumatism (EULAR) [32]. The final questionnaire contained eight questions. The specific questions are described in detail in Table 2.

### Surgical internship evaluation

Clinical surgery internship departments include urology, gastrointestinal surgery, breast surgery, orthopedics, cardiac surgery and thoracic surgery. The evaluation of surgical practice depends mainly on the teacher’s score according to the undergraduate practice evaluation form developed by the first Clinical Medical College of Jinan University. The scoring contents include work attitude, interpersonal communication, clinical case writing, other medical document writing, physical examination, diagnosis and differential diagnosis, treatment plan and prescription, clinical skill operation, language expression ability and self-study ability. There are four scoring options: excellent, good, medium and poor, corresponding to 10 points, 8 points, 6 points and 4 points, respectively, for a total possible score of 100 points. Finally, the average surgery score was recorded according to the student practice manual.

### Objective structure clinical examination

**Fig. 3:**
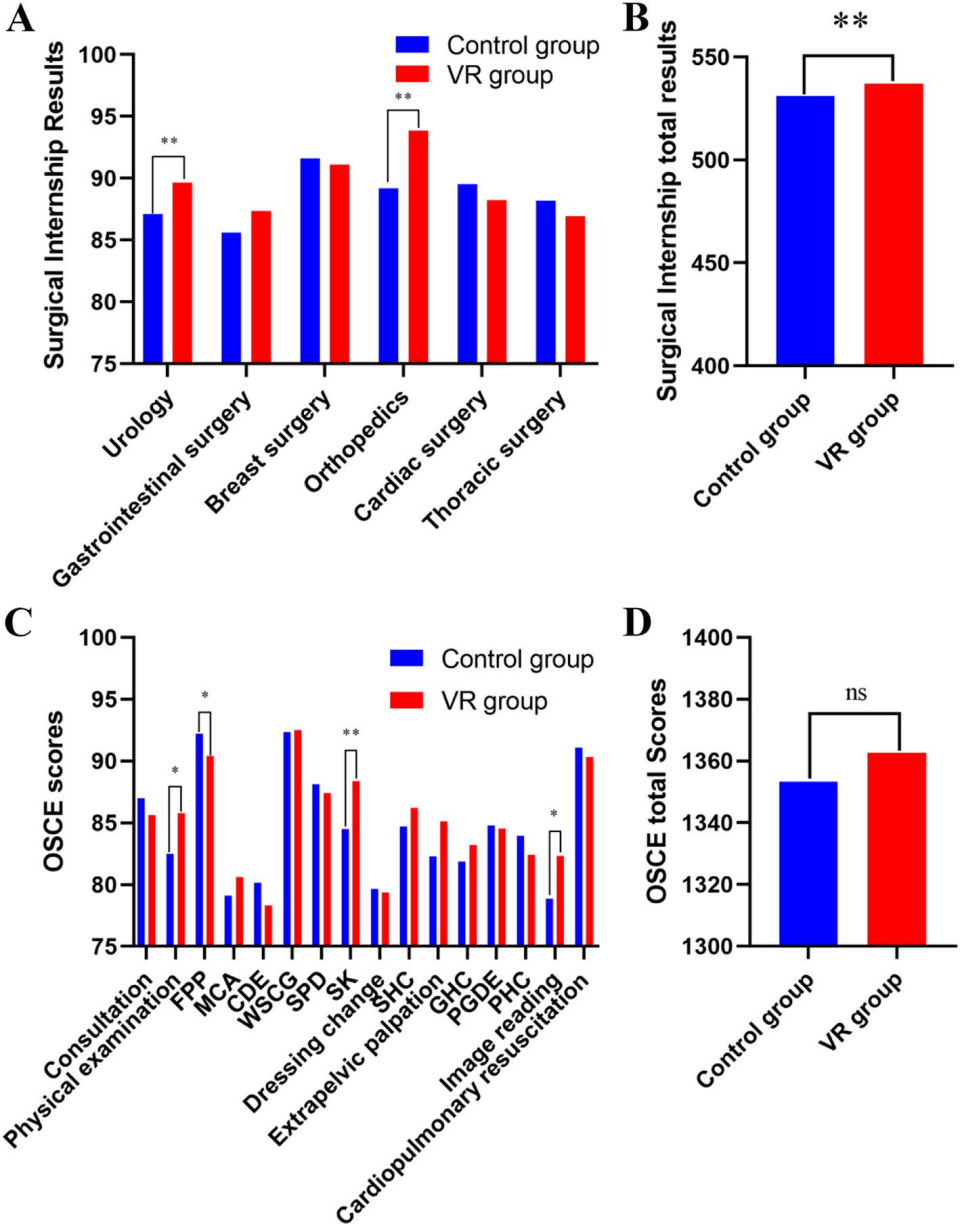
**A**-**B**: Surgical internship results; (**C**-**D**): Objective structure clinical examination scores. FPP: Four puncture procedures; MCA: Medical case analysis; CDE: Cardiopulmonary dummy examination; WSCG: Wearing surgical clothes and gloves; SPD: Skin preparation and draping; SK: Suturing and knotting; SHC: Surgical history collection; GHC: Gynecological history collection; PGDE: Pediatric growth and development evaluation; PHC: Pediatric history collection; ns: not significant

The OSCE was created by the British researcher R. Harden in 1975 [33]. In recent years, it has been widely used as an exclusive assessment, stage assessment and graduation assessment for the standardized training of residents in China. In the study plan of undergraduate medical students set by the First Clinical Medical College of Jinan University, the 16-station comprehensive OSCE examination is taken by medical students after completing their one-year clinical practice. The specific assessment contents are listed in Fig. 3. The final score is the average score for each station, and the OSCE score is derived from the student teaching system.

### Blinding

To eliminate subjective bias induced by knowing students' groups during scoring, the examiners who scored using the global rating scale did not engage in VR teaching activities in the first trial. To eliminate artificial subjective bias in the process, we employed questionnaires to follow up and gather internship and OSCE scores in the follow-up research. The study's existence, much alone the categorization of students, was unknown to departmental practice examiners and OSCE examiners.

### Randomization

A random number table was used to construct a random allocation sequence. The VR and control groups were assigned to the participants using a sequence. The students practiced tendon repair on a synthetic model for the test. One of the two groups was allocated to each participant at random. The long-term follow-up study was divided into groups based on the initial research's categorization.

### Statistics

SPSS version 26.0 software was used for statistical analysis. The Baseline and questionnaire results were compared by means of SPSS average analysis. When determining whether the data were in accordance with a normal distribution, the Kolmogorov-Smirnova technique revealed that variables in questionnaire and consultation, Four puncture procedures (FPP), Cardiopulmonary dummy examination (CDE), Skin preparation and draping (SPD), Suturing and knotting (SK), cardiopulmonary resuscitation in OSCE were not. Consequently, the Mann–Whitney test was used in the data processing. The remaining data were processed in accordance with Levene's test, and it was discovered that the variance was homogenous, thus an independent sample T test was carried out. The difference was statistically significant when the *P* value was less than 0.05. The practice and OSCE results are presented in the "Results" section as (experimental group minus control group SD, *P* value). Some of the results were graphed using GraphPad Prism 8 in the form of a bar chart.

## Results

From August 1st to August 30th in 2021, the Medical College of Jinan University issued a follow-up invitation to 117 participants who had participated in and completed the initial study. Five participants in the VR group did not respond to the follow-up invitation, three participants withdrew from the follow-up study for personal reasons; and one participant in the control group withdrew from the follow-up study for personal reasons. After signing the informed consent form for the follow-up study, all the participants provided the researchers with their clinical practice scores in 6 surgical departments during the one-year clinical practice and their OSCES16 (OSCE score at 16 stations) on the fifth-year graduation examination (Fig. 1). At the same time, all the participants were required to complete a follow-up questionnaire jointly developed by the author and the faculty of the Medical College of Jinan University. After excluding those who were lost to follow-up, we compared the age, gender and undergraduate average scores of the two groups and found no significant differences (*p* > 0.05) (Table 1).

**Table 1:** Baseline characteristics

Characteristics	VR group (*N* = 53)	Control group (*N* = 55)	Total (*N* = 108)	*P* value
Age(mean ± SD, range)	23.98 ± 1.01(22–27)	24.07 ± 0.98(22–26)	24.03 ± 0.99(22–27)	0.633
Gender(male:female)	31:22	23:32	54:54	0.085(male)
Grade point average(mean ± SD)	3.14 ± 0.44	3.01 ± 0.54	3.08 ± 0.50	0.186

### Questionnaire results

As shown in Table 2, when answering the item “strengthened my determination to be a doctor after my internship”, 28 students (52.8%) in the VR group gave a positive response, while 17 students (30.9%) in the control group gave a positive response. The difference was statistically significant (*p* < 0.05). In response to the item “I will habitually review the relevant knowledge of the internship department during the internship”, 18 (33.9%) students in the VR group gave a positive response, while 9 (16.3%) students in the control group gave a positive response. The difference was statistically significant (*p* < 0.05). The differences between the groups in their responses to the other 6 questions were not statistically significant.

**Table 2:** Questionnaire results

	Strongly disagree N(%)	Disagree N(%)	Neutral N(%)	Agree N(%)	Strongly agree N(%)	
1. The experience of internship strengthened my determination to be a doctor						
VR group	6 (11.3)	6 (11.3)	13 (24.5)	24 (45.3)	4 (7.5)	*P* = 0.044
Control group	15 (27.3)	6 (10.9)	17 (30.9)	10 (18.2)	7 (12.7)	
Total	21 (19.4)	12 (11.1)	30 (27.8)	34 (31.5)	11 (10.2)	
2. The internship experience has a great influence on the choice of my department						
VR group	15 (28.3)	4 (7.5)	7 (13.2)	17 (32.1)	10 (18.9)	*P* = 0.657
Control group	13 (23.6)	8 (14.5)	14 (25.5)	9 (16.4)	11 (20)	
Total	28 (25.9)	12 (11.1)	21 (19.4)	26 (24.1)	21 (19.4)	
3. I will habitually review the relevant knowledge of the internship department during the internship						
VR group	0 (0.0)	14 (26.4)	21 (39.6)	14 (26.4)	4 (7.5)	*P* = 0.034
Control group	6 (10.9)	14 (25.5)	26 (47.3)	7 (12.7)	2 (3.6)	
Total	6 (5.6)	28 (25.9)	47 (43.5)	21 (19.4)	6 (5.6)	
4. The experience of internship can help me understand book theory better						
VR group	0 (0.0)	6 (11.3)	13 (24.5)	16 (30.2)	18 (34.0)	*P* = 0.523
Control group	0 (0.0)	9 (16.4)	15 (27.3)	13 (23.6)	18 (32.7)	
Total	0 (0.0)	15 (13.9)	28 (25.9)	29 (26.9)	36 (33.3)	
5. I had a lot of opportunities to get started and learned a lot of knowledge that was not available in books during the internship						
VR group	2 (3.8)	9 (17)	13 (24.5)	19 (35.8)	10 (18.9)	*P* = 0.722
Control group	2 (3.6)	7 (12.7)	18 (32.7)	21 (38.2)	7 (12.7)	
Total	4 (3.7)	16 (14.8)	31 (28.7)	40 (37.0)	17 (15.7)	
6. I tried my best to seize the opportunity of hands-on operation during the internship						
VR group	1 (1.9)	8 (15.1)	24 (45.3)	17 (32.1)	3 (5.7)	*P* = 0.302
Control group	2 (3.6)	16 (29.1)	18 (32.7)	14 (25.5)	5 (9.1)	
Total	3 (2.8)	24 (22.2)	42 (38.9)	31 (28.7)	8 (7.4)	
7. I can handle the relationship between me and my classmates, teachers and nurses very well during the internship						
VR group	2 (3.8)	13 (24.5)	13 (24.5)	19 (35.8)	6 (11.3)	*P* = 0.363
Control group	4 (7.3)	10 (18.2)	11 (20.0)	18 (32.7)	12 (21.8)	
Total	6 (5.6)	23 (21.3)	24 (22.2)	37 (34.3)	18 (16.7)	
8. It is also interesting to write medical records, write doctor's orders and change dressing during the internship						
VR group	9 (17.0)	21 (39.6)	15 (28.3)	7 (13.2)	1 (1.9)	*P* = 0.142
Control group	6 (10.9)	21 (38.2)	12 (21.8)	10 (18.2)	6 (10.9)	
Total	15 (13.9)	43 (38.9)	27 (25.0)	17 (15.7)	7 (6.5)	

### Surgical internship results

The total score for clinical practice in the VR group was better than that in the control group, and the difference was statistically significant (5.95 ± 1.99, *p* < 0.05)(Fig. 3B). As shown in Fig. 3A, the rotational internship scores of the VR group in urology (2.57 ± 0.92, *p* < 0.05) and orthopedics (1.76 ± 0.96, *p* < 0.01) were higher than those of the control group, and the difference was statistically significant. The rotational internship score of the VR group in gastrointestinal surgery (-0.51 ± 0.67, *p* = 0.069) was higher than that of the control group, while the control group scored better than the VR group in breast surgery (4.65 ± 0.92, *p* = 0.450), cardiac surgery (-1.28 ± 0.83, *p* = 0.124) and thoracic surgery (-1.24 ± 0.77, *p* = 0.111). However, the differences were not statistically significant.

### OSCE scores

The statistical findings and participants' overall scores from the OSCES16 are shown in Figs. 3C and 3D. Compared to the control group, the VR group performed somewhat better on average, but the difference was not statistically significant (9.37 ± 5.70, *p* = 0.103). The scores for physical examination (3.30 ± 1.55, *p* < 0.05), suturing and knotting (3.87 ± 1.15, *p* < 0.01) and image reading (3.43 ± 1.59, *p* < 0.05) were higher in the VR group than in the control group, and the difference was statistically significant. The scores for medical case analysis, wearing surgical clothes and gloves, surgical history collection, extrapelvic palpation and gynecological history collection were higher in the VR group than in the control group, but the difference was not statistically significant. We were shocked to find that the control group had higher test scores for the FPP than the VR group did (-1.80 ± 0.80, *p* < 0.05), and the difference was statistically significant. The performance of the control group was better than that of the VR group in consultation (-1.36 ± 1.89, *p* = 0.471), cardiopulmonary dummy examination (-1.86 ± 2.25, *p* = 0.410), skin preparation and draping (*p* = 0.503), dressing change (-0.28 ± 2.00, *p* = 0.890), pediatric growth and development evaluation (-0.25 ± 1.62, *p* = 0.877), pediatric history collection (-1.53 ± 1.30, *p* = 0.243) and cardiopulmonary resuscitation (-0.75 ± 0.69, *p* = 0.282). However, the differences were not statistically significant.

## Discussion

The learning system itself, especially in medical learning, is extremely complex. Studies have shown that a skill will decline completely after 6 to 18 months without repeated practice to strengthen memory [34–36]. For the individual medical student, the ultimate goal of undergraduate education is not to complete the indicators of teaching content in medical theoretical knowledge and practical operation but to cultivate an appropriate learning mode [37].Only an efficient and active learning model can enable medical students to learn continuously and effectively in the face of the dynamic development of their subject knowledge after choosing their future clinical department direction [37, 38]. Hence, we do not propose to test individual skill development by recurrent tendon suturing throughout a one-year long-term follow-up in order to ascertain the long-term impacts of short-term VR simulation learning. Instead, as a complement to the subjective survey, we want to investigate the long-term effects of VR simulation training on the learning style of medical students. To do this, we will use practice assessments, OSCE exams, and development questionnaires.The one-year internship experience is the first time medical students can transform medical knowledge from written learning to clinical application. Clinical study in this year affects the future clinical direction of medical students, initially establishes the clinical communication model and shapes students’ empathy toward patients [39]. We used an expert opinion development questionnaire to understand the subjective evaluation of the clinical practice effect of the medical students in different groups. The results show that the medical students who experienced VR simulation had more firm career choices and took the initiative to review the relevant theoretical knowledge in the process of department rotation. In addition to the subjective evaluation of the students, we also compared the subjective scores from their teachers and found that the total score for surgical internship of the medical students in the VR simulation group was better than that of the control group, and the differences between the groups in urology and orthopedics were statistically significant. The results of this comparison are encouraging because compared with passive knowledge acquisition, problem-based active learning can improve the transformation of knowledge from book learning to clinical application and increase the motivation of self-instruction [34, 40]. The formation of a positive feedback closed loop of the learning model can lead to a better learning experience.

At present, the OSCE is widely used to evaluate the clinical operation ability of clinical medical staff and medical students [41]. It is a comprehensive ability evaluation method that pays equal attention to knowledge, skills and clinical attitude [33]. There was no significant difference between the two groups in the total OSCES16, but the physical examination, suturing and knotting and image reading scores were higher in the VR group than in the control group, and the difference was statistically significant. Additionally, we think that students' performance in the VR group is not any better than that of the control group since they lack the means and resources for future VR practice and other clinical procedures in the research. This result does not show that the VR simulation intervention brought significant learning pattern optimization to the participants in the experimental group, but we have reason to believe that it may act as a process of iterative reinforcement in the nonlinear dynamics. Within nonlinear dynamics systems, output is normally not proportional to input, which shows the sensitivity of the system to initial conditions [42–45]. In other words, a little change in the conditions at the beginning may have a huge impact on the results over a long period of development. The traditional medical teaching mode emphasizes the completion of learning tasks while neglecting the importance of timely feedback in the learning process. The VR simulation teaching mode can provide timely and effective feedback for every learning behavior and improve students’ self-guidance in the learning process. Additionally, VR simulation adds the mechanism of learning task decomposition and visualization of learning content to change students’ cognition of the learning mode [46]. Therefore, in times of network information and the development of science and technology, modern learning needs to not only attach importance to the cultivation of ability but also strengthen learners’ positive emotional experience to enable them to obtain a positive learning mentality [47].

In contrast to conventional tendon repair techniques, learning tendon suturing using a VR simulator greatly improved movement time, process, and procedural knowledge, according to the results of our first research. Despite having higher clinical practice test scores during our extended follow-up, students in the VR group, this outcome was not clearly reflected in the OSCE exam. We think that VR aided teaching has an instant improved impact on teaching quality compared to our first intervention trial and follow-up. However, following the first research, the school was unable to offer resources and channels for VR aided learning in interdisciplinary learning due to a lack of funding and the inadequate support of VR in undergraduate education of medical students. As a consequence, the subsequent interdisciplinary evaluation did not accurately represent this instant improvement.

It has been consistently shown that VR-assisted teaching helps enhance the quality of instant instruction in the clinical medical area. For example, in the field of surgical medicine, VR is currently used for a variety of purposes, including the exploration of potential new surgical plans [48], the design of surgical plans prior to surgery [49], the improvement of clinicians' operational skills and the reduction of the surgical learning curve [50], the direction of patients during postoperative repair [51, 52], etc. Despite the positive outcomes in the aforementioned areas, creating certain unique therapeutic situations demands a significant financial commitment owing to the high cost of VR learning and the complicated and variable clinical scenarios [53]. High-quality studies that assess the advantages of the significant capital expenditure and clinical return brought on by the uniqueness of such application situations are still lacking as of this writing [54]. Pre-clinical training that may be repeated is a more economical use for VR than clinical training, including medical students' undergraduate education. VR may help with immersive learning in the COVID-19 outbreak by making up for the absence of interactive learning and immediate feedback on experience [55, 56]. The use of VR aided teaching models as standard teaching strategies may be possible in the future with the promotion of VR, the lowering of VR use costs, and the investment of VR-related education funding. Without the influence of quick promotion, we will then be able to evaluate the long-term effects of VR supported education on the learning habits of undergraduate medical students and potentially their future professions.

Through the first study experiment, we discovered that VR instruction of tendon suturing increase the level of medical students in terms of movement time, operation procedure, and procedural knowledge. Long-term follow-up revealed that students in the experimental group had a superior future surgical clinical practice and OSCE score after experiencing VR education, which might be due to a new teaching experience gradually influencing their learning style and process. As a result, VR teaching should be included into the learning process of undergraduate clinical courses, not only for the instruction of tendon suture but also as an auxiliary teaching tool to run through the whole learning process of clinical courses as far as feasible, if situations permit.

### Limitation

First, individuals in this curriculum are required to do a one-year hospital internship after their theoretical coursework. This finding may not be relevant to the teaching model in other nations as this is the clinical medicine teaching paradigm in China. Second, the results of this research have certain limitations since the VR Achilles tendon rehabilitation teaching intervention approach used in this study did not include other therapeutic disciplines. Thirdly, participant performance in future clinical trial rotations and OSCE evaluations were the key criteria used to evaluate the long-term follow-up of this investigation. As a single intervention in a VR teaching mode, the intervention may not have been strong enough to completely rule out the impact of certain unidentified elements in the participants' later learning. Multidisciplinary VR educational interventions are recommended. Fourthly, there are just a few individuals in this single-center research. More prospective trials will be required in the future to investigate the usefulness of VR simulation in medical instruction.

## Conclusion

The results of the one-year long-term follow-up showed that medical students who had exposure to the VR teaching mode exhibited more steadfast career pursuit and a propensity to engage in active learning, as well as receiving higher instructor ratings throughout the surgical clinical practice phase. Meanwhile, the VR teaching mode may help medical students perform better on the OSCE in the areas of physical examination. In the study of nonlinear dynamics to cultivate a good learning model for medical students, the VR teaching model is expected to become an effective and stable initial sensitive element.

## Supplementary Information

**Additional file 1: Table S1.** Presents the specific teaching situation of experimental group and control group in the initial study.

## Data Availability

On reasonable request, we agree to get data for this research by contacting the corresponding author.

## Abbreviations

VR: virtual reality
OSCE: Objective Structure Clinical Examination
GPA: Grade Point Average
EULAR: European League Against Rheumatism
OSCES16: OSCE score at 16 stations
FPP: Four puncture procedures
MCA: Medical case analysis
CDE: Cardiopulmonary dummy examination
WSCG: Wearing surgical clothes and gloves
SPD: Skin preparation and draping
SK: Suturing and knotting
SHC: Surgical history collection
GHC: Gynecological history collection
PGDE: Pediatric growth and development evaluation
PHC: Pediatric history collection
