# Supplementary material for: Researching the application of virtual reality in medical education: one-year follow-up of a randomized trial
Source: BMC Med Educ. 2023 Jan 3;23:3. doi: 10.1186/s12909-022-03992-6 (PMC9808681; doi:10.1186/s12909-022-03992-6)
Supplement: Supplementary file 1 — Additional file 1: Table S1. Presents the specific teaching situation of experimental group and control group in the initial study. [file 12909_2022_3992_MOESM1_ESM.docx]

|  | **VR Group** | **Control Group** |
| --- | --- | --- |
| **Lectures**  **(Total 8 hours)** | ✓ | ✓ |
| **Practical Class**  **(Total 6 hours)** | ✓ | ✓ |
| **Guided PowerPoint Review** |  | ✓ |
| **VR+PC Practice** | ✓ |  |
| **VR+PC Assessment** | ✓ |  |

Table S1: Illustration showing different parts of the training were undertaken by each group.
